# Neuronal Correlates of Functional Coupling between Reach- and Grasp-Related Components of Muscle Activity

**DOI:** 10.3389/fncir.2017.00007

**Published:** 2017-02-21

**Authors:** Shashwati Geed, Martha L. McCurdy, Peter L. E. van Kan

**Affiliations:** ^1^Motor Systems Physiology Laboratory, Department of Kinesiology, University of Wisconsin–Madison, MadisonWI, USA; ^2^Department of Rehabilitation Medicine, Georgetown University Medical Center, WashingtonDC, USA

**Keywords:** reach to grasp, cerebellum, magnocellular red nucleus, coordination, nucleus interpositus

## Abstract

Coordinated reach-to-grasp movements require precise spatiotemporal synchrony between proximal forelimb muscles (shoulder, elbow) that transport the hand toward a target during reach, and distal muscles (wrist, digit) that simultaneously preshape and orient the hand for grasp. The precise mechanisms through which the redundant neuromuscular circuitry coordinates reach with grasp, however, remain unclear. Recently, Geed and Van Kan (2016) demonstrated, using exploratory factor analysis (EFA), that limited numbers of global, template-like transport/preshape- and grasp-related muscle components underlie the complexity and variability of intramuscular electromyograms (EMGs) of up to 21 distal and proximal muscles recorded while monkeys performed reach-to-grasp tasks. Importantly, transport/preshape- and grasp-related muscle components showed invariant spatiotemporal coupling, which provides a potential mechanism for coordinating forelimb muscles during reach-to-grasp movements. In the present study, we tested whether ensemble discharges of forelimb neurons in the cerebellar nucleus interpositus (NI) and its target, the magnocellular red nucleus (RNm), a source of rubrospinal fibers, function as neuronal correlates of the transport/preshape- and grasp-related muscle components we identified. EFA applied to single-unit discharges of populations of NI and RNm neurons recorded while the same monkeys that were used previously performed the same reach-to-grasp tasks, revealed neuronal components in the ensemble discharges of both NI and RNm neuronal populations with characteristics broadly similar to muscle components. Subsets of NI and RNm neuronal components were strongly and significantly crosscorrelated with subsets of muscle components, suggesting that similar functional units of reach-to-grasp behavior are expressed by NI and RNm neuronal populations and forelimb muscles. Importantly, like transport/preshape- and grasp-related muscle components, their NI and RNm neuronal correlates showed invariant spatiotemporal coupling. Clinical and lesion studies have reported disruption of coupling between reach and grasp following cerebellar damage; the present results expand on those studies by identifying a neuronal mechanism that may underlie cerebellar contributions to spatiotemporal coordination of distal and proximal limb muscles during reaching to grasp. We conclude that finding similar functional units of behavior expressed at multiple levels of information processing along interposito-rubrospinal pathways and forelimb muscles supports the hypothesis that functionally related populations of NI and RNm neurons act synergistically in the control of complex coordinated motor behaviors.

## Introduction

Reach-to-grasp movements require precisely coordinated activation of shoulder, elbow, wrist, and digit muscles such that while shoulder and elbow muscles transport the hand toward a target during reach, wrist and digit muscles preshape and orient the hand for grasp. Psychophysical studies of reach-to-grasp movements have demonstrated functional coupling between reach and grasp such that the wrist follows a largely bell-shaped velocity profile during reach and attains peak velocity at approximately 70% of the transport trajectory (Jeannerod, 1984). Peak wrist velocity during the transport phase coincides in time with attainment of maximal grip aperture (i.e., distance between the thumb and index finger), which characterizes hand opening prior to closing the hand in anticipation of grasp (Jeannerod, 1981, 1984). Furthermore, perturbation of target location, which directly impacts the reach, also influences grasp (Paulignan et al., 1991b; Roy et al., 2006), and perturbation of target size or orientation (Paulignan et al., 1991a, 1997; Roy et al., 2006), which directly impacts grasp, also influences the reach. Recently, Geed and Van Kan (2016) demonstrated, using exploratory factor analysis (EFA), invariant spatiotemporal coupling between transport/preshape- and grasp-related components of forelimb muscle activity in monkeys performing reach-to-grasp tasks, consistent with the observed functional coupling between reach and grasp movements.

Reaching to grasp critically depends on cerebellar function. Cerebellar damage causes a specific breakdown in coupling of reach and grasp movement components (Bastian and Thach, 1995; Bastian et al., 1996, 2000; Mason et al., 1998; Lang and Bastian, 1999; Cooper et al., 2000; Rand et al., 2000; Zackowski et al., 2002). Although cerebellar output targets many, if not all, neural structures involved in movement production, its most direct influences are exerted via the pathway from nucleus interpositus (NI), the sole output of intermediate cerebellum, to the magnocellular red nucleus (RNm). RNm receives its dominant input from NI (Humphrey and Rietz, 1976; Kennedy et al., 1986; Houk et al., 1988), and RNm neurons terminate as rubrospinal fibers on spinal interneurons, or directly on motoneurons that innervate digit muscles (Kuypers et al., 1962; Lawrence and Kuypers, 1968a,b; Kuypers, 1982; McCurdy et al., 1987; Holstege et al., 1988; Ralston et al., 1988; McCurdy et al., 1992). In keeping with influences on distal limb muscles, rubrospinal fibers show promise for prehensile recovery following stroke (Carmel et al., 2013; Takenobu et al., 2014), and following experimental lesions of the pyramidal tract in monkeys (Belhaj-Saif and Cheney, 2000). Therefore, studying the role of interposito-rubrospinal (NI-RNm) circuitry in the control of coordinated reach-to-grasp movements is not only important for understanding cerebellar function in general but is also important for potential neuromodulation and rehabilitation post-stroke, which is in line with recent investigations in other subcortical pathways (Baker, 2011; Bradnam et al., 2013; Cunningham et al., 2015).

Although a mechanistic understanding of how NI-RNm circuits contribute to control of spatiotemporal coordination of reach and grasp movement components has remained elusive, several lines of evidence indicate that NI-RNm circuitry is important to this process. First, forelimb NI and RNm neurons discharge consistently and at high rates when monkeys reach to grasp objects (NI: Gibson et al., 1994; Van Kan et al., 1994; Van Kan and McCurdy, 2001, 2002a,b), RNm: (Gibson et al., 1994; Van Kan et al., 1994; Van Kan and McCurdy, 2001, 2002a,b), and the high discharge rates of NI and RNm neurons are associated with coordinating the hand in the context of whole-limb reaching movements (Miller et al., 1993; Van Kan et al., 1994; Van Kan and McCurdy, 2001). Second, behavioral studies indicate that lesioning or inactivating NI or RNm (NI: Mason et al., 1998; Cooper et al., 2000); RNm: (Sybirska and Gorska, 1980; Gibson et al., 1994) profoundly and specifically affect coordination of reach and grasp movement components. Third, anatomical investigations have demonstrated that NI-RNm circuitry projects (through rubrospinal pathways) to interneuronal and motoneuronal pools that innervate forelimb muscles crucial for coordinated reach-to-grasp movements (Kuypers et al., 1962; Lawrence and Kuypers, 1968a,b; Kuypers, 1982; McCurdy et al., 1987; Holstege et al., 1988; Ralston et al., 1988; McCurdy et al., 1992). The combined results of electrophysiological, behavioral, and anatomic studies support strongly the hypothesis that NI-RNm circuitry serves as a potential neuronal correlate of reaching to grasp.

The primary objective of the current study was to test whether EFA, applied to ensembles of single-unit discharges of populations of NI and RNm neurons recorded while the same monkeys that were used previously (Geed and Van Kan, 2016) performed the same reach-to-grasp tasks, would reveal neuronal components with characteristics broadly similar to the muscle components identified. Our results demonstrate that EFA did indeed reveal 5–7 NI or RNm neuronal components in each monkey, which, while explaining a large proportion of the variance in the ensemble discharges of the NI and RNm neurons recorded, showed significant correlations with the transport/preshape- or grasp-related muscle components identified. Importantly, in both NI and RNm populations, invariant spatiotemporal coupling between transport/preshape- and grasp-related neuronal components closely resembled the coupling observed between transport/preshape- and grasp-related muscle components. The results of the present study significantly strengthen our hypothesis that the combined output from NI-RNm circuitry reflects a neuronal correlate of the functional coupling between reach and grasp movement components.

## Materials and Methods

Two rhesus macaques (Macaca mulatta, male, 7–10 kg) were trained to perform reach-to-grasp movements. Animal care and experimental procedures complied with the United States Public Health Service Policy on Humane Care and Use of Laboratory Animals, conformed to the National Institutes of Health, “Guide for the Care and Use of Laboratory Animals,” and were approved by the Institutional Animal Care and Use Committee of the University of Wisconsin – Madison. A more detailed description of behavioral paradigms, surgical implantation of EMG electrodes, and data collection procedures has been provided in earlier publications (Van Kan and McCurdy, 2001, 2002a). Brief reports of these results have appeared in abstract form (Geed et al., 2011, 2013).

### Experimental Protocol

The two monkeys (*W. B*) performed reach-to-grasp tasks with their right forelimb while seated upright in a primate chair with their backs and feet supported. A neck collar and waist plate loosely restrained the animals while seated. The reaching forelimb and head were unrestrained. The animals were trained to reach and grasp a cereal reward (Kellogg’s^®^ *Froot Loops*^®^ Cereal, thickness: ∼6 mm; diameter: ∼19 mm) using either a precision or whole-hand grasp from a target assembly located in the parasagittal plane through the shoulder of the animal’s reaching limb, 56° above the horizontal plane through the shoulder. The cereal reward was dispensed in a horizontally oriented narrow slot (height: 6 mm, width: 25 mm, depth: 25 mm), which necessitated apposition of the index finger and thumb in a precision grasp, or a 50-ml glass beaker (clear, diameter: 32 mm, tilted at a 45° angle toward the animal), which required concerted use of all digits in a whole-hand grasp.

A typical reach-to-grasp trial began with the animal holding a handle at the waist for a variable inter-trial interval of 3-5 *s*, which minimized anticipatory muscle activity prior to movement onset. During the inter-trial interval, the animal received water reward for holding the handle steady at the waist. To initiate a reaching movement, a computer-controlled air cylinder dispensed the cereal reward into either the beaker or narrow slot, and a light-emitting diode (LED) next to the receptacle with the cereal reward lit up cueing the animal to initiate its reach-to-grasp movement as well as instructing whether to reach to the beaker (for whole-hand grasp) or slot (for precision grasp). Upon illumination of the LED, the monkey released the handle at the start location, reached toward the target assembly, retrieved the cereal reward from the beaker or slot as instructed, returned its hand to the mouth to eat the cereal reward, and finally, moved its hand back to the starting position to grasp the handle. The next trial was initiated following the variable inter-trial interval. The cereal reward was presented in the narrow slot or beaker in a pseudorandom fashion, controlled by custom software running in LabVIEW (National Instruments, LabVIEW 7.1).

### Data Recording and Preprocessing

Behavioral event time data, intramuscular EMGs, and single-unit discharges from RNm and NI neurons were recorded in both monkeys. EMG recording sessions were carried out separately from the single-unit recording sessions although EMGs and neuronal data were recorded from the same two monkeys performing the same reach-to-grasp tasks.

#### Behavioral Event Markers

Behavioral event times were recorded with contact sensors on the handle at the starting location, on the slot, and on the beaker’s rim at the target locations. Reach onset and offset were defined as the times of breaking contact with the handle and making contact with the slot or beaker, respectively. Grasp onset and offset were defined as the times of making and breaking contact with the slot or beaker, respectively. Behavioral event times were used to normalize the durations of transport and grasp intervals over trials and to align trials.

#### Intramuscular Electromyograms (EMGs)

The complete sets of muscles implanted in both monkeys, as well as the frequency of recording sessions in a given muscle is shown in **Table 1**. We recorded activity from 14 (monkey *W*) and 20 (monkey *B*) forelimb muscles in sets of 9 muscles/recording session on different days in close succession while the monkeys performed precision and whole-hand reach-to-grasp tasks. EMG signals were rectified, integrated (time constant: 10 ms), band-pass filtered (30 Hz – 3 kHz), and digitized at 167 Hz by A/D computer inputs (CED 1401 *plus*, Cambridge Electrical Design). Rectified, integrated, and band-pass filtered EMG signals were low-pass filtered using a fourth-order zero-lag Butterworth filter with a cut-off frequency of 15 Hz.

**Table 1:** Forelimb muscles recorded in each monkey.

	Muscle	Abbreviation	Recording frequency	
			
			Monkey *W*	Monkey *B*
Digits	Extensor digitorum communis	EDC	7/7	3/5
	Extensor digitorum two and three	ED23		3/5
	Extensor digitorum four and five	ED45	2/7^∗^	1/5
	Flexor digitorum superficialis	FDS	6/7	1/5
	Flexor digitorum profundus	FDP	3/7	2/5
	Extensor pollicis longus	EPL		1/5
	Abductor pollicis longus	APL	7/7	2/5
	Palmaris longus	PL	3/7	3/5
Wrist	Extensor carpii radialis	ECR	6/7	3/5
	Extensor carpii ulnaris	ECU		3/5
	Flexor carpii radialis	FCR	6/7	3/5
	Flexor carpii ulnaris	FCU	2/7	4/5
Elbow	Brachioradialis	BR		2/5
	Biceps	BIC	2/7	2/5
	Triceps	TRI	4/7	2/5
Shoulder	Acromion deltoid	AcDLT	5/7	2/5
	Spino deltoid	SpDLT	4/7	2/5
	Cleido deltoid	ClDLT		2/5
	Pectoralis	PEC	4/7	2/5
	Teres major	TM		2/5
	Latissimus dorsii	LAT	2/7	


*EMGs were recorded in 12 sessions (1 session/day) over a 2–3-week period. Seven sessions were conducted in monkey *W*, 5 in monkey *B*. In a given session, EMGs of combinations of 9 muscles were recorded simultaneously. Numerical entries indicate recording frequency, i.e., (number of sessions in which EMGs of a given muscle were recorded) / (total number of sessions). ^∗^EMGs of ED45 in monkey *W* were not included in the analyses because of technical difficulties. EMGs, intramuscular electromyograms.*

#### Neural Recordings

**FIGURE 1:**
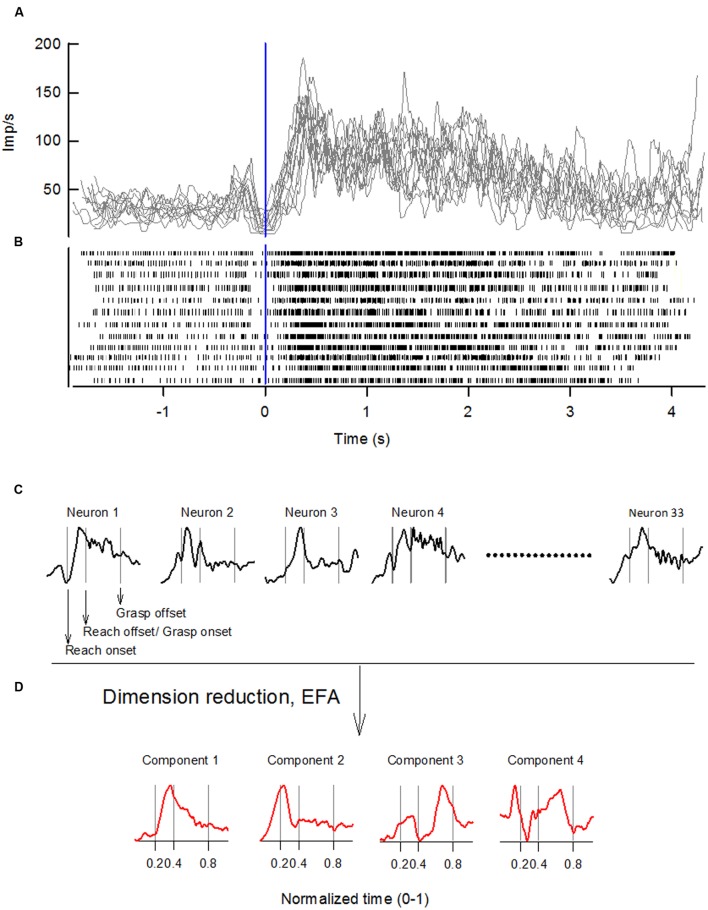
**Sequence of steps for extracting neuronal components by applying EFA to ensembles of single-unit discharges of NI and RNm neurons.** **(A)** Records of discharge frequency of a single RNm neuron, computed from interspike intervals, are overplotted for 12 representative reach-to-grasp trials of the precision task in monkey *W*. Records are aligned on reach onset (Time 0, vertical line). **(B)** Spike-raster of neural discharge showing the same 12 reach-to-grasp trials as in **(A)**. **(C)** Records of discharge frequency of each neuron were time normalized, standardized, and trial-averaged to yield a dataset of the population of neurons with *n* = 1, 2, … n (here *n* = 33). EFA with *varimax* factor rotation was applied to the correlation matrix of this neuronal dataset to derive a low-dimensional representation of the data. **(D)** A limited number of neuronal components was retained following EFA. Behavioral event times are indicated by vertical lines in **(C,D)**: reach onset, reach offset/grasp onset, grasp offset respectively.

Discharges of forelimb RNm and NI neurons were recorded with epoxylite-coated tungsten microelectrodes (exposed tip of microelectrodes: 15–25 μm) as monkeys performed the precision or whole-hand reach-to-grasp tasks. We recorded from 33 and 34 forelimb RNm neurons in monkey *W* and monkey *B*, respectively; and from 30 and 48 forelimb NI neurons in monkey *W* and monkey *B*, respectively. Microelectrodes were inserted through the dura mater with a microdrive (Narishige MO-97), modified to include a stainless-steel guide tube assembly, which allowed the microelectrode tip to traverse the dura without being damaged. The microdrive was covered by a lightweight cylinder (diameter: 100 mm), which prevented the animal access to the microdrive. Single-unit discharges were monitored visually on an oscilloscope, filtered (half-amplitude band-pass at 100 Hz and 10 kHz ± 3dB), and fed into a window discriminator circuit that produced a standard pulse for each action potential. Discriminated pulses were used as computer clock triggers to collect interspike intervals with 100-μs precision. **Figure 1B** shows representative spike trains recorded from an individual RNm neuron over 12 reach-to-grasp trials of the precision task in monkey *W.*

#### Data Preprocessing

Each neuron’s discharge frequency was computed from the interspike interval record by averaging the neuron’s discharge rate over consecutive 6-ms periods taking into account fractional interspike intervals. Task-related modulations in discharge rate during individual trials were quantified by calculating the average discharge rate over a 100-ms window that was moved, 6 ms at a time, between the times of reach onset and grasp offset. This created a record of the neuron’s discharge frequency throughout the reach-to-grasp trial. **Figure 1A** shows records of discharge frequency computed from neural spikes recorded from a single RNm neuron recorded over 12 reach-to-grasp trials. Additional time intervals of 500 ms preceding reach onset, and 250 ms following grasp offset were also included to account for neural activity before reach onset and during the early part of the hand’s return to the mouth.

#### Outlier Removal

Trials with outlier durations of the transport or grasp phase were removed using Rosner’s Many Outliers Procedure (Rosner, 1983). A trial was removed if the duration of either the transport or grasp phase was determined to be an outlier. Most outlier trials had unusually long durations. The number of reach-to-grasp trials removed from EMGs ranged from 0/40 (0%) to 6/44 (13.6%) in monkey *B*, and from 1/87 (1.1%) to 6/84 (7.1%) in monkey *W.* The number of reach-to-grasp trials removed from RNm data ranged from 8/223 (3.6%) in monkey *B* to 21/187 (11.2%) in monkey *W*. The number of outlier trials removed in NI data ranged from 40/741 (5.4%) in monkey *B* to 21/209 (10%) in monkey *W*.

#### Trial Alignment

The transport and the grasp phase of each trial was time normalized to the mean duration of transport or grasp phase in a given monkey. Time-normalized trials were aligned on reach onset and averaged across trials. Thus, all precision reach-to-grasp data from different EMG recording sessions were averaged across trials to give a single dataset with time-normalized, trial-averaged values of the activation amplitudes of 14 muscles in monkey *W* (or 20 muscles in monkey *B*). Similarly, there was a single dataset with time-normalized, trial-averaged values of the 33 RNm neurons, and 30 NI neurons in monkey *W* (or 34 RNm neurons, and 48 NI neurons in monkey *B*). Each of the EMG, RNm, and NI datasets were standardized to have zero mean and unit standard deviation as required for subsequent factor analysis. **Figure 1C** shows the time-normalized, trial-averaged, standardized activity of a representative set of RNm neurons.

### Data Analysis

#### Exploratory Factor Analysis on Muscle and Neuronal Data

Exploratory factor analysis with varimax factor rotation was applied to the correlation matrices of time-normalized, trial-averaged, standardized EMGs, RNm, and NI data separately to derive a low-dimensional representation of the data. The low-dimensional representation retained the variance of muscle or neuronal data while allowing for computationally simpler comparisons between groups of 14 or 20 muscles, and the combined output of 33 or 34 RNm neurons, and 30 or 48 NI neurons in monkey W and monkey B, respectively.

The goal of applying EFA is to represent “D” number of muscles (or neurons in case of neuronal components) as a linear combination of N components with N < D such that:

$$m(t)=\overset{N}{\underset{i=1}{\Sigma }}c_{i}(t)w_{i}$$

Here *m(t)* is a D-dimensional vector that specifies the activation of each muscle (or neuron) at time t. *c_i_(t)*, referred to as the component’s temporal scaling coefficient is a time-varying scaling coefficient for the *i*-th component. *w_i_* (D × N matrix) represents the weighting coefficients of the i-th component, the relative strength of activation of the muscle (or neuron) in a given component. The weighting coefficients range between +1 and -1, with strong increased activation of a muscle (or neuron) in the component represented by a value close to +1 and strong decreased activation represented by a value close to -1. *c_i_(t)*, which is a (N × t) matrix, represents the time-varying temporal scaling coefficient of the i-th component throughout the reach-to-grasp movement. **Figure 1D** shows the temporal scaling coefficients from a representative set of RNm neuronal components.

The number of muscle or neuronal components to retain following EFA was based on *(1)* Kaiser criteria (Kaiser, 1974), and *(2)* Scree plot of the extracted components and amounts of variance explained (Cattell, 1966). The combined criteria ensured that the components retained in the factor analysis contributed to meaningful interpretation of muscle and neuronal activity in the context of reaching to grasp, and captured a sizable amount of variance in the data whereas components that accounted for relatively small contributions to the variance of the collected sample were excluded. EFA was carried out using SPSS version 20 (SPSS IBM, New York).

#### Characterizing the Functional Contributions of Muscle Components

Detailed criteria for characterizing a muscle component as predominantly transport/preshape- or grasp-related have been described previously (Geed and Van Kan, 2016). Briefly, a muscle component was characterized as either transport/preshape- or grasp-related based on the combination of two criteria: *(1)* combinations of muscles showing weighting coefficient values greater than 0.4 (w_i_ > 0.4), and *(2)* timing of maximal contribution of the temporal scaling coefficient during the reach-to-grasp trial. Cross-referencing the temporal scaling coefficients with weighting coefficients is critical to determine the functional contribution of a given muscle component because the weighting coefficients reflect the relative ratios of activation of a combination of muscles in the component, and the temporal scaling coefficients reflect the activation profile of the combination of muscles in a component over time. Taken together, the weighting and temporal scaling coefficients indicate a component’s functional contribution during reach-to-grasp movements. Based on these criteria, cross-referencing showed that muscle components 1 and 2 contributed predominantly during the transport/preshape phase in both monkeys. Muscle components 3 and 4 (4 only in monkey *W*) contributed predominantly during grasp in both monkeys.

#### Comparison of Neuronal and Muscle Components

The cross-correlation function was used to compare temporal scaling coefficients of muscle components with RNm and NI neuronal components. We hypothesized that if NI and RNm discharges represent neuronal correlates of coordinated reach-to-grasp muscle activity, we would find broad similarities between the temporal scaling coefficients of muscle, RNm, and NI components. Pair-wise cross-correlation magnitudes between each of the muscle-RNm, and muscle-NI component pairs were computed using Matlab^®^ (MathWorks). The highest cross-correlation magnitude within a predefined -50 to +30 normalized time-bin window defined the best-matching muscle-neuronal component pair. The next highest cross-correlation defined the next best-matching muscle-neuronal component pair and so on, until there were no more unpaired components left in either the neuronal or muscle datasets.

Negative cross-correlation lags signify that a neuronal component occurs earlier than the muscle component on the normalized time scale, whereas positive lags signify that a neuronal component lags the muscle component on the normalized time scale. The loss of the absolute durations of individual trials was a drawback of time normalizing each trial for further analysis to compare the forelimb muscle activity and neuronal activities in low-dimensional space; however, we predefined a relative duration of -50 to +30 bins as the relevant time-window for meaningful neuronal-muscle signal interaction. This duration was chosen in accordance with Miller et al. (1993), who have reported that relatively analogous time lag windows (-150 to 200 ms) capture approximately 85% of the significant cross-correlation peaks between activity of single RNm neurons and EMGs recorded during free-form forelimb movements in monkeys. Before time normalization, each bin represented 6-ms worth of EMG or neuronal data, and so our predefined time window of -50 to +30 bins of normalized time captures the majority of the neuronal-muscle interactions of interest. We considered relatively short positive lags as meaningful too because small positive lags may signify parallel descending inputs to muscle components, to which NI-RNm components contribute only partially or at particular times during the entire duration of the reach-to-grasp movement.

Significance tests for cross-correlations are not well defined. Therefore, Monte Carlo simulations were used to determine the probability that the cross-correlation peak at a given time lag would occur by chance. The muscle component signal was randomly shuffled, and cross-correlations were computed between this randomly shuffled muscle component signal and the neuronal component signal. This process was repeated 10,000 times for each muscle-neuronal component pair to generate a distribution of peak cross-correlation values between the 10,000 randomly shuffled muscle components and the neuronal component. The 0.5th and 99.5th percentiles for this distribution served as the upper and lower bounds of significance for the cross-correlation (i.e., *p* ≤ 0.01). If peak cross-correlations between muscle and neuronal components occurred due to chance, values would fall between the upper and lower bounds; however, statistically significant cross-correlation values would be outside the confidence interval allowing us to reject the null hypothesis that muscle and neuronal components were uncorrelated. Significance testing of the cross-correlations using Monte Carlo simulations was performed offline using custom programs in MATLAB^®^ (MathWorks).

#### Temporal Coupling between Transport/Preshape- and Grasp-Related Neuronal Correlates

Transport/preshape- and grasp-related muscle components show invariant spatiotemporal coupling during reach-to-grasp movements irrespective of the type of grasp or the target location in the workspace (Geed and Van Kan, 2016). The time of peak activation of the transport/preshape-related muscle component occurs simultaneously with the time of peak slope of activation of the grasp-related muscle component. In the present study, we tested the hypothesis that NI and RNm components represent the neuronal correlates of the invariant spatiotemporal coupling between transport/preshape- and grasp-related muscle components. Slope (*M*) of the grasp-related component was computed using the following equation,

$$M=\frac{\left(y_{2}-y_{1}\right)}{\left(x_{2}-x_{1}\right)}$$

where (*x_1,_ y_1_*), and (*x_2,_ y_2_*) are points on the grasp-related component of interest. A paired-samples *t*-test was used to determine if the NI and RNm neuronal correlates of the transport/preshape- and grasp-related muscle components showed similar functional coupling as reported for muscle components.

## Results

Two monkeys performed reach-to-grasp tasks that required either a precision or whole-hand grasp to retrieve cereal reward. This report is based on single-unit discharges of task-related forelimb NI and magnocellular red nucleus (RNm) neurons (NI: *n* = 30 and *n* = 48, RNm: *n* = 33 and *n* = 34, in monkey *W* and monkey *B*, respectively), and intramuscular EMGs of forelimb muscles (*n* = 14 and *n* = 20 in monkey *W* and monkey *B*, respectively, **Table 1**). Data from the RNm neurons have been included in previous reports (Van Kan and McCurdy, 2001, 2002a,b). EMGs included in the present report are a subset of those included in Geed and Van Kan (2016). EMGs were recorded during reach-to-grasp movements to only one target location (“up”) of the four locations included in the previous report.

**FIGURE 2:**
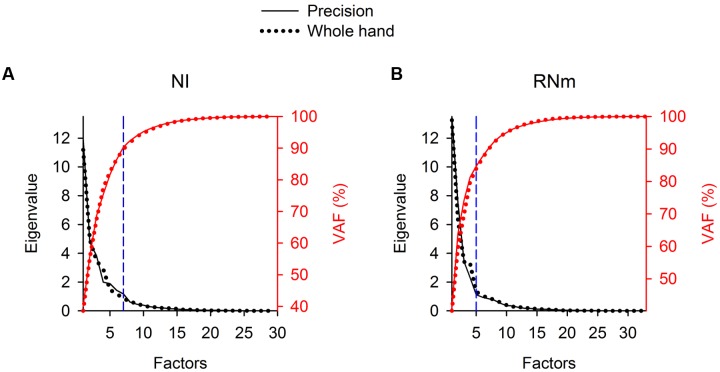
**Eigenvalues and percent variance accounted for (%VAF) of NI** **(A)** and RNm **(B)** neuronal components in Monkey *W*. Scree plots (left ordinates, black) illustrate the progressive decrease in eigenvalues of extracted neuronal components as the number of components selected increases. The first 7 (in NI) or 5 (in RNm) components had eigenvalues > 1, which fulfills the Kaiser criteria for retaining factors as components. The “elbow” in the Scree plots (dashed, vertical blue lines) marks the cut off for retaining the first 7 (NI) or 5 (RNm) factors as neuronal components. Plots of %VAF (right ordinates, red) as a function of the number of factors demonstrate that the first 7 (or 5 in RNm) accounted for >85% of the variance in NI or RNm data. Solid lines show data for the precision task, dotted lines show data for the whole-hand task.

For each task condition, EFA was used to determine whether a limited number of components is able to explain a large amount of variance in the ensemble discharges of the NI and RNm neurons of our sample. Scree plots (**Figure 2**, left ordinates, black) show the progressive decrease in eigenvalues of extracted factors as the number of factors selected increases. The first 7 factors (NI, **Figure 2A**) or 5 factors (RNm, **Figure 2B**) extracted had eigenvalues > 1, which fulfills the Kaiser criteria for retaining factors as components (see Materials and Methods). Plots of percent variance accounted for (% VAF) as a function of the number of factors (**Figure 2**, right ordinates, red) demonstrate that the first 7 components (NI, **Figure 2A**) or 5 components (RNm, **Figure 2B**) accounted for >85% of the variance in the ensemble discharges of NI and RNm neurons for both precision (solid lines) and whole-hand tasks (dotted lines). **Table 2** summarizes % VAF by NI and RNm neuronal components, and muscle components. Percent VAF by muscle components was taken from Geed and Van Kan (2016). **Supplementary Table 1** shows cumulative variances accounted for by each of the neuronal and muscle components in the two monkeys during precision and whole-hand tasks. In summary, EFA revealed that 7-5 NI and RNm neuronal components accounted for >85% of the variance in ensemble discharges of the NI and RNm neurons of our sample, and 4–6 muscle components accounted for >85% of the variance in EMGs of the forelimb muscles we sampled.

**Table 2:** Variance accounted for by NI, RNm, and muscle components.

	Monkey *W*		Monkey *B*	
		
	Whole-hand	Precision	Whole-hand	Precision
NI	89.7 (7)	90.4 (7)	92.9 (6)	93.4 (7)
RNm	87.0 (6)	85.0 (5)	91.9 (6)	89.9 (5)
Muscle	90.1 (4)	86.9 (4)	93.0 (6)	91.8 (5)


*Values represent percent variance accounted for (% VAF) by the number of components (n) in parentheses.*

### Neuronal Correlates of Muscle Components

In a recent study, Geed and Van Kan (2016) reported that muscle components extracted using EFA were functionally aligned with transport/preshape- or grasp-related aspects of reach-to-grasp movements. In the following sections, we demonstrate similarities in characteristics of the NI, RNm, and muscle components we identified. Pairwise cross-correlations quantified similarities between temporal scaling coefficients *c_i_*(*t*) of the best-matching NI and RNm neuronal components and those of their corresponding transport/preshape- or grasp-related muscle components. Of note, each muscle component contributed throughout the reach-to-grasp movement; however, components were characterized as mainly transport/preshape- or grasp-related in order to simplify the expression of muscle activity of up to 21 forelimb muscles, and to evaluate relations between muscle components and neuronal components extracted from ensemble discharges of NI and RNm neurons.

#### Neuronal Correlates of Transport/Preshape-Related Muscle Components

**FIGURE 3:**
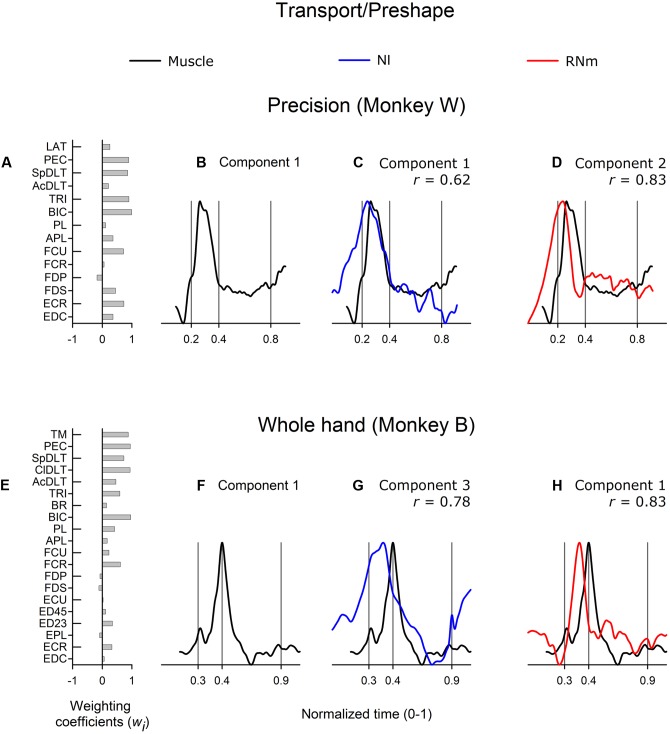
**Best-matching NI and RNm neuronal correlates of transport/preshape-related muscle component 1.** **(A,E)** Weighting coefficients of transport/preshape-related muscle component 1 in precision **(A)** and whole-hand **(E)** task in monkey *W* and monkey *B*, respectively. **(B,F)** Temporal scaling coefficients of transport/preshape-related muscle component 1. Temporal scaling coefficients shown in **(B,F)** specify activation of all muscles included in the muscle component according to their corresponding weighting coefficients shown in **(A,E)**, respectively. **(C,G)** Temporal scaling coefficients of the NI components that best matched with transport/preshape-related muscle component 1 shown in **(B,F)**, respectively. **(D,H)** Temporal scaling coefficients of the RNm components that best matched with transport/preshape-related muscle component 1 shown in **(B,F)** respectively. Cross-correlation magnitude between NI or RNm and muscle components is indicated by *r*. Behavioral event times are indicated by vertical lines: reach onset, reach offset/grasp onset, grasp offset respectively. Ordinate scales are arbitrary but uniform throughout. Time scale is normalized (0–1) across neuronal and EMG reach-to-grasp data to allow neuronal-muscle comparisons.

**Figure 3** shows correlations between the best-matching NI and RNm neuronal components and transport/preshape-related muscle component 1. Muscle component 1 increased activity at or prior to reach onset, attained peak amplitude during the transport/preshape phase prior to (**Figure 3B**, monkey *W*) or at the time the hand made contact with the target (**Figure 3F**, monkey *B*), and sharply decreased activity during the latter half of the transport/preshape phase (**Figure 3B**) or early in the grasp phase (**Figure 3F**). Component 1 was characterized by strong contributions from proximal muscles with coactivation of wrist and digit muscles (**Figure 3A**, monkey *W*; **Figure 3E**, monkey *B*).

The best-matching NI components (NI component 1 in monkey *W*; NI component 3 in monkey *B*) were strongly correlated with transport/preshape-related muscle component 1 in both animals (**Figure 3C**, *r* = 0.62, precision task in monkey *W*; **Figure 3G**, *r* = 0.78, whole-hand task in monkey *B*). The best-matching NI components explained 23.2 and 19.9% of the variance in the ensemble discharges of the 30 and 48 forelimb NI neurons in monkey *W* and monkey *B*, respectively. The best matching RNm components (component 2 in monkey *W*, precision task; and component 1 in monkey *B*, whole-hand task) were strongly correlated with transport/preshape-related muscle component 1 (**Figure 3D**, *r* = 0.83, precision task in monkey *W*; **Figure 3H**, *r* = 0.83, whole-hand task in monkey *B*). RNm component 2 explained 24.3% and component 1 explained 26.8% of the variance in the ensemble discharges of the 33 and 34 forelimb RNm neurons in monkey *W* and monkey *B*, respectively.

**FIGURE 4:**
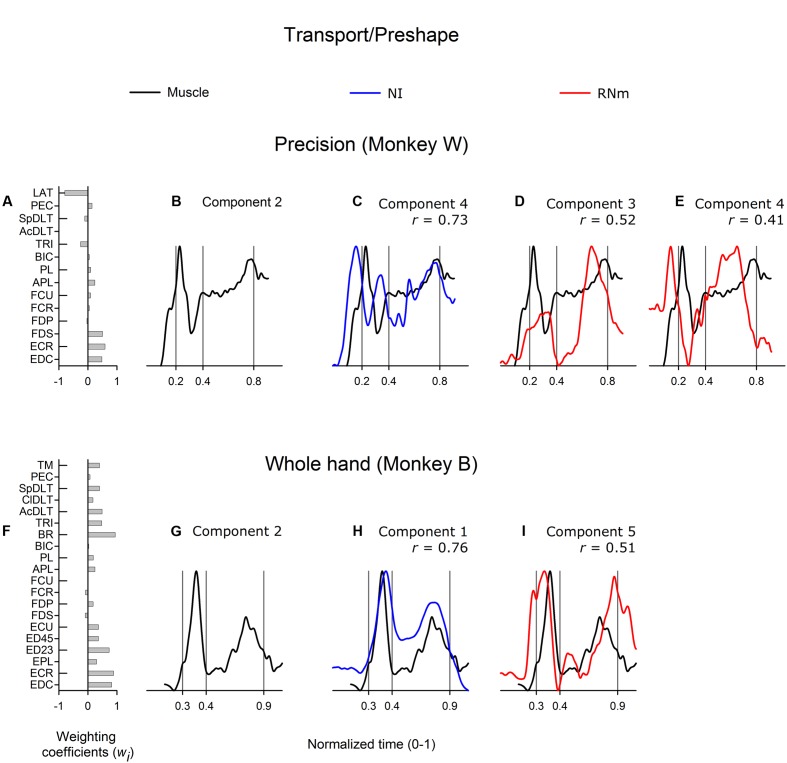
**Best-matching NI and RNm neuronal correlates of transport/preshape-related muscle component 2.** **(A,F)** Weighting coefficients of transport/preshape-related muscle component 2 in precision **(A)** and whole-hand **(F)** task in monkey *W* and monkey *B*, respectively. **(B,G)** Temporal scaling coefficients of transport/preshape-related muscle component 2. Temporal scaling coefficients shown in **(B,G)** specify activation of all muscles included in the muscle component according to their corresponding weighting coefficients shown in **(A,F)**, respectively. **(C,H)** Temporal scaling coefficients of the NI components that best matched with transport/preshape-related muscle component 2 shown in **(B,G)**, respectively. **(D,E,I)** Temporal scaling coefficients of the RNm components that best matched with transport/preshape-related muscle component 2 shown in **(B,G)** respectively. Format as in **Figure 3**.

**Figure 4** shows correlations between the best-matching NI and RNm neuronal components and transport/preshape-related muscle component 2. Muscle component 2 (**Figure 4B**, monkey *W*; **Figure 4G**, monkey *B*) attained peak activation amplitude during the transport/preshape phase and sharply decreased activity around grasp onset. A second, relatively smaller peak of activation was attained in the latter third of the grasp phase or early during the return phase when the hand moved back to the mouth to ingest the food reward. Muscle component 2 showed combined activity of shoulder, wrist, and digit muscles (**Figure 4A**, monkey *W*; **Figure 4F**, monkey *B*).

The best matching NI neuronal components (NI component 4 in monkey *W*; NI component 1 in monkey *B*) were strongly correlated with transport/preshape-related muscle component 2 (**Figure 4C**, *r* = 0.73, precision task in monkey *W*; **Figure 4H**, *r* = 0.76, whole-hand task in monkey *B*). The best-matching NI components explained 11.3%, and 29.2% of the variance in the ensemble discharges of the 30 and 48 forelimb NI neurons recorded in monkey *W* and monkey *B*, respectively. The best matching RNm components (RNm components 3 and 4 in monkey *W*; RNm component 5 in monkey *B*) were moderately correlated with transport/preshape-related muscle component 2 (**Figure 4D**, *r* = 0.52; **Figure 4E**, *r* = 0.41). The RNm neuronal components explained 12.4% and 8.7% of the variance in the ensemble discharges of the 33 RNm neurons in monkey *W.* RNm component 5 in monkey *B* was moderately correlated with transport/preshape-related muscle component 2 (**Figure 4I**, *r* = 0.51, whole-hand task) and explained 11.1% of the variance in the ensemble discharges of the 34 RNm neurons in monkey *B*.

In summary, a considerable amount of variance in the ensemble discharges within both the NI and RNm populations was directed toward transport/preshape-related aspects of reach-to-grasp movements in both animals during performance of both tasks. In addition, the transport/preshape-related NI and RNm neuronal components showed strong to moderate correlations with the transport/preshape-related muscle components.

#### Neuronal Correlates of Grasp-Related Muscle Components

**FIGURE 5:**
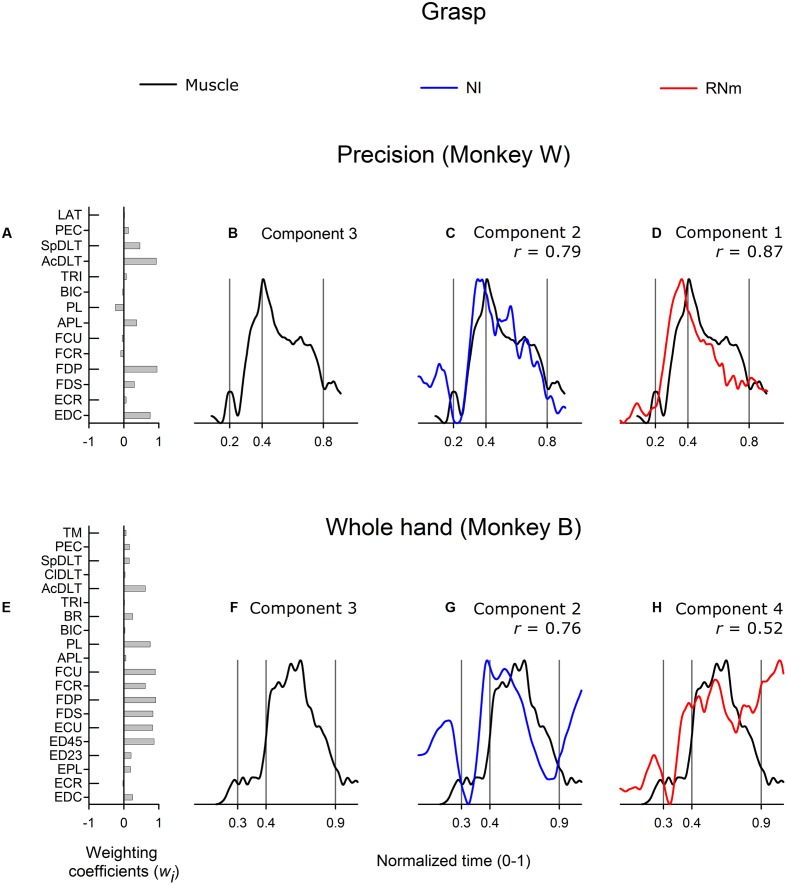
**Best-matching NI and RNm neuronal correlates of grasp-related muscle component 3.** **(A,E)** Weighting coefficients of grasp-related muscle component 3 in precision **(A)** and whole-hand **(E)** task in monkey *W* and monkey *B* respectively. **(B,F)** Temporal scaling coefficients of grasp-related muscle component 3. Temporal scaling coefficients shown in **(B,F)** specify activation of all muscles included in the muscle component according to their corresponding weighting coefficients shown in **(A,E)**, respectively. **(C,G)** Temporal scaling coefficient of the NI components that best matched with grasp-related muscle component 3 shown in **(B,F)**, respectively. **(D, H)** Temporal scaling coefficient of the RNm components that best matched with grasp-related muscle component 3 shown in **(B,F)**, respectively. Format as in **Figure 3**.

**Figure 5** shows correlations between the best-matching RNm and NI neuronal components and grasp-related muscle component 3. Grasp-related muscle component 3 attained peak amplitude early in the grasp phase, near the time the hand contacted the target, and remained active at a relatively high amplitude throughout the first two-thirds (**Figure 5B**, precision task in monkey *W*) or first half (**Figure 5F**, whole-hand task in monkey *B*) of the grasp phase. Grasp-related muscle component 3 included combinations of proximal and distal muscles: EDC, FDP, AcDLT, and SpDLT in monkey *W* (**Figure 5A**, precision task) and various wrist and digit flexor and extensor muscles in combination with BR in monkey *B* (**Figure 5E**, whole-hand task).

The best matching NI neuronal components (NI component 2 in both monkeys) were strongly correlated with grasp-related muscle component 3 in both monkeys (**Figure 5C**, *r* = 0.79, precision task in monkey *W*; **Figure 5G**, *r* = 0.76, whole-hand task in monkey *B*). The best matching NI neuronal components accounted for 19.6 and 20.3% of the variance in the ensemble discharges of the 30 and 48 NI neurons in monkey *W* and monkey *B*, respectively. The best matching RNm neuronal component in monkey *W* (RNm component 1) was strongly correlated with grasp-related muscle component 3 (**Figure 5D**, *r* = 0.87), whereas correlations were moderate in monkey *B* (**Figure 5H**, *r* = 0.52). The best matching RNm components accounted for 32.8 and 12.8% of the variance in the ensemble discharges of the 33 and 34 forelimb RNm neurons in monkey *W* and monkey *B*, respectively. Thus, grasp-related components of NI and RNm neuronal populations showed strong to moderate correlations with grasp-related muscle component 3 in both monkeys but explained relatively variable amounts of the variance in the ensemble discharges of RNm versus NI neurons.

#### Higher-Order Muscle Components

**FIGURE 6:**
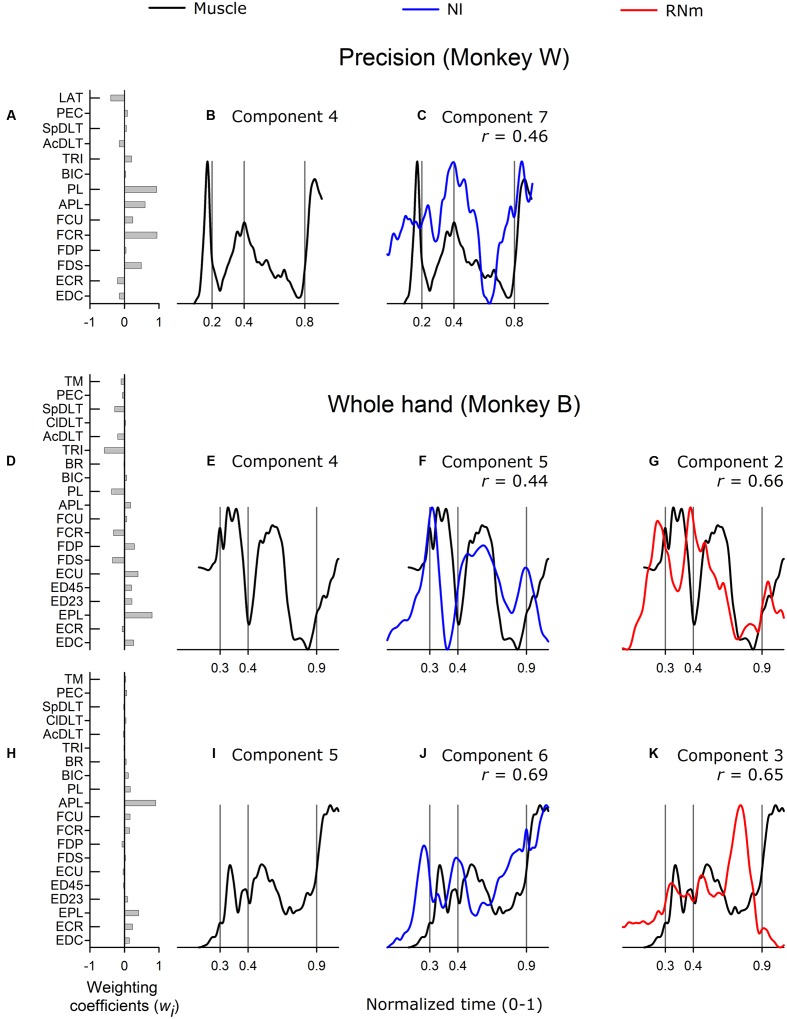
**Best-matching NI and RNm neuronal correlates of higher-order muscle components.** **(A)** Weighting coefficients of grasp-related muscle component 4 in precision task in monkey *W.* **(B)** Temporal scaling coefficient of muscle component 4 specifies activation all muscles included in the muscle component according to their corresponding weighting coefficients shown in **(A)**. **(C)** Temporal scaling coefficient of the NI component that best matched with grasp-related muscle component 4 shown in **(A,B)**. **(D,E)** and **(H,I)** Show weighting coefficients and temporal scaling coefficients of muscle components in whole-hand task in monkey *B.* **(F,G)** and **(J,K)** Temporal scaling coefficient of best-matching NI and RNm neuronal components in the whole-hand task in monkey *B*. Format as in **Figure 3**.

**Figure 6** illustrates correlations between best matching neuronal components and higher-order muscle components. Grasp-related muscle component 4 was identified in both precision and whole-hand tasks in monkey W and included wrist and digit flexors (**Figure 6A**). The temporal scaling coefficient (**Figure 6B**) was characterized by a sharp and brief peak in the pre-movement phase when the monkey grasped the device handle prior to reach onset. A second, smaller peak occurred at grasp onset, and a third rapid increase in amplitude occurred during the return phase when the monkey, having grasped the cereal reward, started to move the hand toward the mouth in order to ingest the cereal reward. The best-matching NI component was moderately correlated (**Figure 6C**, *r* = 0.46) and accounted for 2.5% of the variance in ensemble discharges of the 30 forelimb NI neurons in monkey *W*. No best-matching RNm neuronal correlate of grasp-related muscle component 4 was identified.

Muscle components 4 and 5 in monkey *B* were not formally classified as transport/preshape- or grasp-related given our predefined criteria for selection of a factor as a muscle component (eigenvalue > 1, scree-plot criteria; Geed and Van Kan, 2016). Nevertheless, these components were compared with neuronal components extracted from the ensemble discharges of NI or RNm neurons because previous studies have reported that higher-order muscle components carry meaningful control information (Brochier et al., 2004). Muscle component 4 and 5 were characterized by strong contribution of EPL (**Figure 6D**) or APL (**Figure 6H**) and small contributions from many other forelimb muscles. Muscle components 4 and 5 accounted for relatively small amounts of variance in EMGs (9.6 and 5.8% for components 4 and 5, respectively). The best-matching NI component to muscle component 4 (**Figure 6F**) was moderately correlated (*r* = 0.44) and accounted for 7.8% of the variance in the ensemble discharges of the 48 NI neurons in monkey *B*. The best matching RNm component to muscle component 4 (**Figure 6G**) was strongly correlated (*r* = 0.66) and accounted for 17.4% of the variance in the ensemble discharges of the 34 RNm neurons in monkey *B*. The best matching neuronal correlates to muscle component 5 showed strong correlations (NI, **Figure 6J**, *r* = 0.69; RNm, **Figure 6K**, *r* = 0.65), and accounted for 7.2 and 17.4% of the variance in the ensemble discharges of the 48 NI and 34 RNm neurons in monkey *B*, respectively. Thus, NI and RNm neuronal correlates were identified even for higher-ordered muscle components, which explained relatively small amounts of variance in EMGs.

In summary, a considerable amount of variance in the ensemble discharges within both the NI and RNm populations sampled is directed toward both transport/preshape-related and grasp-related aspects of reach-to-grasp movements in both animals during performance of both tasks. The results of cross-correlating NI and RNm neuronal components and muscle components support the view that the ensemble discharges of populations of NI and RNm neurons are part of the neural substrate underlying coordinated reach-to-grasp behaviors.

### Temporal Coupling between Transport/Preshape- and Grasp-Related Neuronal and Muscle Components

**FIGURE 7:**
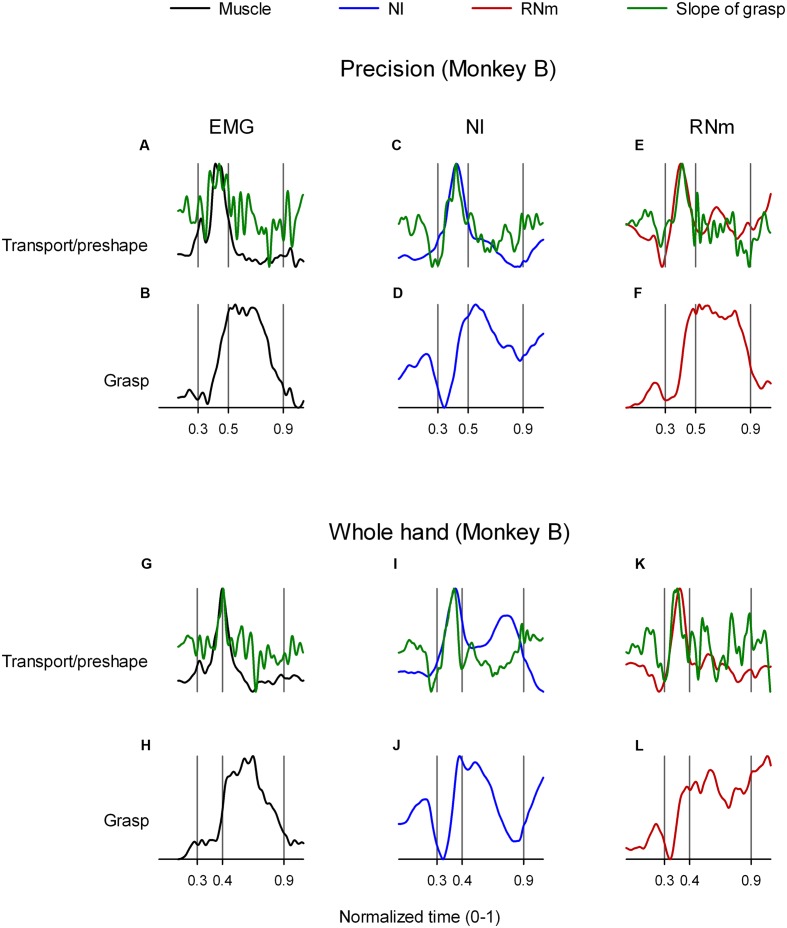
**Temporal coupling between transport/preshape- and grasp-related muscle and neuronal components.** **(A,C,E)** and **(G,I,K)** show temporal scaling coefficients of transport/preshape-related muscle (black), NI (blue), and RNm (red) components in monkey *B* during precision **(A,C,E)** and whole-hand **(G,I,K)** tasks. **(B,D,F)** and **(H,J,L)** show temporal scaling coefficients of the grasp-related components that correspond to the transport/preshape-related components shown in **(A,C,E)** and **(G,I K)** respectively. Slope of grasp-related components (green) is overplotted on the temporal scaling coefficient of the corresponding transport/preshape-related components to highlight synchronicity between peak activation of transport/preshape-related components and peak slope of corresponding grasp-related components. For example, **(C,D)** illustrate temporal coupling between transport/preshape- and grasp-related NI neuronal components in monkey *B* during performance of the precision task. Temporal scaling coefficients of the transport/preshape- and the corresponding grasp-related component are plotted (blue) in **(C,D)**, respectively. The slope of the grasp-related component was calculated (see Materials and Methods) and is overplotted (green) on the temporal scaling coefficients of the preshape/transport-related component in *C.*

Geed and Van Kan (2016) recently demonstrated that transport/preshape- and grasp-related muscle components are spatiotemporally coupled such that peak activation of the transport/preshape-related component is precisely synchronized in time with the peak slope of activation of its corresponding grasp-related component. **Figures 7A,B** (precision task) and **Figures 7G,H** (whole-hand task) illustrate the temporal synchrony between transport/preshape-related muscle component 1 (**Figures 7A,G**, black) and grasp-related muscle component 3 (**Figures 7B,H**) in monkey *B*. Records of the slope of activation amplitudes of grasp-related muscle components as a function of time were calculated (Methods) and are overplotted (**Figures 7A,G**, green) on activation amplitudes of the corresponding transport/preshape-related muscle component (**Figures 7A,G**, black). Analogous to the invariant temporal coupling between transport/preshape- and corresponding grasp-related muscle components, **Figures 7C,D** (precision task) and **Figures 7I,J** (whole-hand task) show similar spatiotemporal coupling between NI neuronal correlates of transport/preshape-related and corresponding grasp-related muscle components. **Figures 7E,F** (precision task) and **Figures 7K,L** (whole-hand task) show similar spatiotemporal coupling between RNm neuronal correlates of transport/preshape-related and corresponding grasp-related muscle components.

**Table 3** summarizes the times of peak amplitude of transport/preshape-related components and the times of peak slope of activation of the corresponding grasp-related components in NI, RNm, and muscle domains for precision and whole-hand tasks in both monkeys. Consistent with our hypothesis, the neuronal correlates of transport/preshape- and grasp-related muscle components are temporally coupled such that times of peak activation of transport/preshape-related NI and RNm neuronal correlates are precisely synchronized with times of peak slope of activation of the corresponding grasp-related NI and RNm neural correlates. A paired-samples *t*-test showed no significant difference [*t*(11) = -0.64, *p* = 0.54] between the times of peak activation of the transport/preshape-related components and the times of peak slope of activation of the corresponding grasp-related components in NI, RNm, and muscle domains.

**Table 3:** Temporal coupling between transport/preshape- and grasp-related NI, RNm, and muscle components.

	Whole-hand		Precision	
		
	T_transport_	T_grasp_	T_transport_	T_grasp_
**Monkey *W***				
NI	0.29	0.28	0.31	0.32
RNm	0.31	0.32	0.25	0.27
Muscle	0.29	0.28	0.28	0.29
**Monkey *B***				
NI	0.39	0.39	0.40	0.40
RNm	0.38	0.35	0.38	0.39
Muscle	0.44	0.44	0.39	0.42


*Values represent normalized time (0–1). T_transport_, time of peak activation of transport/preshape-related component. T_grasp_, time of peak slope of activation of corresponding grasp-related component.*

In summary, the results presented in this section provide evidence for invariant spatiotemporal coupling between NI and RNm neuronal correlates of transport/preshape- and grasp-related muscle components that is qualitatively and quantitatively similar to the invariant spatiotemporal coupling observed between corresponding muscle components.

## Discussion

Our results demonstrate that neuronal components, extracted from the ensemble of single-unit discharges of populations of NI and RNm neurons recorded while monkeys performed reach-to-grasp tasks, are strongly, consistently, and systematically correlated with transport/preshape- and grasp-related muscle components. Importantly, as was found for transport/preshape- and grasp-related muscle components (Geed and Van Kan, 2016), both NI and RNm neuronal correlates show invariant spatiotemporal coupling, suggesting that during reach-to-grasp movements, ensembles of neurons within NI and RNm distribute their variances along the functional dimensions of transport/preshape or grasp such that the timing of activation of transport/preshape is invariantly coupled with the timing of activation of grasp. These results add mechanistic insight to data from clinical and lesion studies that have reported decoupling between reach and grasp following cerebellar lesions. Overall, the results provide strong support for the hypothesis that interposito-rubrospinal (NI-RNm) circuitry underlies spatiotemporal coordination of complex reach-to-grasp behaviors.

### Similar Units of Behavior Are Expressed in Neural and Muscle Signals

Our result of finding similar functional units of behavior at three successive levels (NI, RNm, and EMGs) of information processing in the NI-RNm circuitry supports the view that intermediate cerebellar output engages synergistic groups of neurons and muscles to coordinate reach-to-grasp movements. The neuronal components we identified provide a weighting of each neuron’s contribution to the ensemble average based on the neuron’s correlation pattern with all other neurons in the dataset. Each neuron contributes portions of its variance to each of the neuronal components in a neuron-specific, weighted fashion, and linear combinations of these neural weights, scaled by the neuronal component’s temporal scaling coefficients, account for a large proportion of the variance in the ensemble discharges of the neuronal population. Neuronal components thus distill motor control information distributed across a population of neurons into limited numbers of discrete motor command signals.

The EFA-based neuronal components we identified are comparable with previous reports on information processing in M1 and RNm. For instance, Miller et al. (1993) proposed that groups of mutually correlated RNm neurons may contribute control signals in muscle space that are summed in the spinal cord, in line with the neuronal component framework where linear combinations of a few neuronal components may specify the activation parameters of synergistic groups of muscles. M1 and RNm neurons have been shown to process information in muscle-based “functional linkage vectors (FLVs)” (Miller et al., 1993; Holdefer and Miller, 2002). Krouchev and Drew (2013) demonstrated that a limited number of sparse muscle synergies explained substantial proportions of the variance in cat forelimb EMGs, and they proposed, based on their previously recorded data in cat motor cortex during the same behaviors (Drew, 1993; Drew et al., 1996, 2008), that multiple populations of pyramidal tract neurons (PTNs) may be involved in regulating each of the specific sparse muscle synergies.

Neuronal components, FLVs, and sparse synergies share the conceptual premise that populations of neurons contribute to activity of functionally-related muscle groups; however, the different algorithms underlying cluster and factor analyses result in small differences. FLVs involve correlated activity between individual neurons and groups of muscles, which are then clustered to identify the sub-populations of neurons that share relatively similar correlation patterns with groups of muscles. In contrast, EFA-based neuronal components provide a weighting of each neuron’s contribution to the population average according to its pattern of correlation with all other neurons in the sample. Our approach of identifying a neural basis of muscle components thus adopts a relatively direct population-based view of interrelationships within the neuronal ensemble in order to identify how groups of neurons may specify activation parameters to control groups of muscles as synergistic units. Further, clustering of FLVs leads to a model in which each neuron contributes exclusively to a given muscle “synergy” because clustering algorithms group variables (neurons) so that the variance within a cluster is minimized while the variance between clusters is maximized. In our EFA-based approach, each neuron contributes portions of its variance toward each of the components in the model in a weighted fashion. The same set of neurons may thus apportion their variances differently across each of the components to give rise to a flexible and diverse range of motor behaviors. This mechanism is consistent with recent findings from ICMS-evoked cortical “synergies” where each unit (neuron) appeared to also encode synergies other than the “most evoked synergy” (Overduin et al., 2014, 2015).

Our finding of limited numbers of neuronal components underlying the ensemble activity of forelimb NI and RNm neurons is significant because it demonstrates for the first time that NI-RNm circuitry may specify motor commands in the same low-dimensional space as found for muscle components underlying naturally-occurring reach-to-grasp behaviors in monkeys. In particular, finding neuronal correlates of higher-order muscle components (shown in **Figure 6**) indicates that similar functional units of reach-to-grasp behaviors are isolated from ensemble discharges of neurons and EMGs even if the muscle components account for relatively small amounts of variance in the EMGs. Thus, higher-order components of reach-to-grasp muscle activity may transmit meaningful information with respect to behavior, which is consistent with the findings of Brochier et al. (2004) that higher-order muscle components were able to discriminate between grasp types in their reach-to-grasp task. Evidence consistent with our findings has also come from transcranial magnetic stimulation (TMS) (Gentner and Classen, 2006) and intracortical microstimulation (ICMS) (Overduin et al., 2012, 2014, 2015) of various motor cortical sites in human and non-human primates, respectively. TMS- or ICMS-evoked digit movements demonstrate similar muscle synergies as identified during naturally-occurring digit movements, such as grasping either imagined (Gentner and Classen, 2006) or differently shaped objects (Overduin et al., 2012). Whereas the TMS- or ICMS-based studies support the hypothesis of building blocks underlying voluntary motor control in the motor cortex, our framework of neuronal components in NI-RNm circuitry supports and expands previous findings by proposing a mechanism and additional substrates by which neuromotor circuitry may organize functional building blocks of behavior.

### Cerebello-Rubrospinal Circuitry and Spatiotemporal Coupling of Reach and Grasp

Transport/preshape- and grasp-related muscle components show invariant temporal coupling with each other such that the time at which the temporal scaling coefficient of a given transport/preshape-related muscle component attains peak activity during transport coincides exactly with the time of peak slope of activation of the corresponding grasp-related muscle component (Geed and Van Kan, 2016). This relationship was preserved in monkeys preforming reach-to-grasp movements irrespective of the type of grasp or target location in the workspace, indicating that the temporal coupling may reflect a central mechanism to coordinate transport/preshape-related muscle activity with grasp-related muscle activity. The findings in the present study support and extend this hypothesis. NI and RNm neuronal correlates of transport/preshape- and grasp-related muscle components were temporally coupled such that the times of peak activation of transport/preshape-related neuronal correlates coincided with the times of peak slope of activation of corresponding grasp-related neuronal correlates. The invariant spatiotemporal coupling was not a function of the orthogonal relationship between components as a result of the varimax rotation in our factor analysis algorithm because non-orthogonal rotation of extracted factors (promax rotation) yielded similar temporal scaling coefficients and coupling, suggesting that the spatiotemporal coupling did not depend on specific rotation of extracted factors.

Muscle components reflect synchronous activation of functionally-related groups of muscles as synergistic units. Muscles included in a given component are activated in precise ratios with respect to each other as defined by the component weighting coefficients *w_i_*. The temporal scaling coefficients specify the activation amplitude of a muscle component throughout the movement. Thus, a muscle component gives rise to precise spatiotemporal coupling between muscles included in the component. Temporal coupling between muscle components thus enforces a higher-level of structure on how combinations of muscles included in the respective components are activated. Temporal coupling between activation waveform of transport, and slope of activation waveform of grasp, implies that neuromotor circuitry activates transport/preshape-related muscle components in direct relation to corresponding grasp-related muscle components.

Significantly, strong to moderate correlation between neuronal and muscle components suggests that populations of NI and RNm neurons process information about synergistic groups of forelimb muscles. This view is supported by findings that mossy fiber *inputs* to intermediate cerebellum show high joint specificity (Van Kan et al., 1993a,b) whereas even at the hierarchical level of single NI neurons, which represents the sole *output* of intermediate cerebellum, information processing pertains to combinations of multiple forelimb joints (Van Kan et al., 1993b). The transformation of highly joint-specific cerebellar afferent input through mossy fibers into broad whole-limb based neuromotor information in cerebellar output through NI is consistent with our results that the ensemble output from NI reflects control signals for coordinating groups of transport/preshape- and grasp-related muscles synergistically.

Further, NI projects to two extra-cerebellar targets: indirectly, via ventrolateral (VL) thalamus, to primary motor cortex (Rispal-Padel and Latreille, 1974; Jorntell and Ekerot, 1999) and directly to contralateral RNm neurons (Flumerfelt et al., 1973; Asanuma et al., 1983a,b). RNm neurons give rise to descending projections which, via the rubrospinal tract (Shinoda et al., 1977; Shinoda et al., 1982; Holstege et al., 1988; Shinoda et al., 1988; Pong et al., 2002), target interneurons and motoneurons of forelimb muscles (McCurdy et al., 1987; Holstege et al., 1988; Ralston et al., 1988). Converging evidence from behavioral neurophysiology in NI and RNm (Van Kan et al., 1994; Gibson et al., 1996; Van Kan and McCurdy, 2001, 2002a,b), lesion studies in NI (Mason et al., 1998; Cooper et al., 2000; Martin et al., 2000; Johnson et al., 2001), and clinical data from patients with cerebellar lesions (Bastian and Thach, 1995; Bastian, 1997, 2002; Bastian et al., 2000; Zackowski et al., 2002; Morton and Bastian, 2004, 2007) support the view that NI-RNm circuitry plays an important role in the coordination of proximal and distal forelimb muscles during whole-limb multi-joint movements. Thus, physiologically, NI-RNm circuitry is well placed to specify coordination between synergistic groups of proximal and distal forelimb muscles, consistent with the invariant spatiotemporal coupling identified between the functional domains of transport/preshape and grasp.

## Conclusion

The results provide evidence for the hypothesis that ensemble discharges of forelimb neurons in NI and RNm function as neuronal correlates of synergistic, functionally-related groups of proximal and distal forelimb muscles in monkeys performing natural reach-to-grasp movements. The NI and RNm neuronal correlates of transport/preshape- and grasp-related muscle components show precise and invariant spatiotemporal coupling, which is essential for coordinated activation of forelimb muscles during reach-to-grasp behaviors.

## Author Contributions

SG, MM, and PvK contributed to design, acquisition, analysis, and interpretation of data for this work. SG wrote the draft for the manuscript and prepared all figures. SG, MM, and PvK revised and edited the manuscript. SG, MM, and PvK approved the final draft of the manuscript, and agree to be accountable for all aspects of the work.

## Conflict of Interest Statement

The authors declare that the research was conducted in the absence of any commercial or financial relationships that could be construed as a potential conflict of interest.

## References

[B1] Asanuma C. Thach W. T. Jones E. G. (1983a). Brainstem and spinal projections of the deep cerebellar nuclei in the monkey, with observations on the brainstem projections of the dorsal column nuclei. *Brain Res. Rev.* 5 299–322. 10.1016/0165-0173(83)90017-6 6189563

[B2] Asanuma C. Thach W. T. Jones E. G. (1983b). Distribution of cerebellar terminations and their relation to other afferent terminations in the ventral lateral thalamic region of the monkey. *Brain Res.* 286 237–265. 10.1016/0165-0173(83)90015-2 6189561

[B3] Baker S. N. (2011). The primate reticulospinal tract, hand function and functional recovery. *J. Physiol.* 589 5603–5612. 10.1113/jphysiol.2011.215160 21878519 PMC3249036

[B4] Bastian A. J. (1997). Mechanisms of ataxia. *Phys. Ther.* 77 672–675. 9184691 10.1093/ptj/77.6.672

[B5] Bastian A. J. (2002). Cerebellar limb ataxia: abnormal control of self-generated and external forces. *Ann. N. Y. Acad. Sci.* 978 16–27. 10.1111/j.1749-6632.2002.tb07552.x 12582038

[B6] Bastian A. J. Martin T. A. Keating J. G. Thach W. T. (1996). Cerebellar ataxia: abnormal control of interaction torques across multiple joints. *J. Neurophysiol.* 76 492–509. 8836239 10.1152/jn.1996.76.1.492

[B7] Bastian A. J. Thach W. T. (1995). Cerebellar outflow lesions: a comparison of movement deficits resulting from lesions at the levels of the cerebellum and thalamus. *Ann. Neurol.* 38 881–892. 10.1002/ana.410380608 8526460

[B8] Bastian A. J. Zackowski K. M. Thach W. T. (2000). Cerebellar ataxia: torque deficiency or torque mismatch between joints? *J. Neurophysiol.* 83 3019–3030. 10805697 10.1152/jn.2000.83.5.3019

[B9] Belhaj-Saif A. Cheney P. D. (2000). Plasticity in the distribution of the red nucleus output to forearm muscles after unilateral lesions of the pyramidal tract. *J. Neurophysiol.* 83 3147–3153. 10805709 10.1152/jn.2000.83.5.3147

[B10] Bradnam L. V. Stinear C. M. Byblow W. D. (2013). Ipsilateral motor pathways after stroke: implications for non-invasive brain stimulation. *Front. Hum. Neurosci.* 7:184 10.3389/fnhum.2013.00184 PMC3647244 23658541

[B11] Brochier T. Spinks R. L. Umilta M. A. Lemon R. N. (2004). Patterns of muscle activity underlying object-specific grasp by the macaque monkey. *J. Neurophysiol.* 92 1770–1782. 10.1152/jn.00976.2003 15163676

[B12] Carmel J. B. Kimura H. Berrol L. J. Martin J. H. (2013). Motor cortex electrical stimulation promotes axon outgrowth to brain stem and spinal targets that control the forelimb impaired by unilateral corticospinal injury. *Eur. J. Neurosci.* 37 1090–1102. 10.1111/ejn.12119 23360401 PMC3618589

[B13] Cattell R. B. (1966). The scree test for the number of factors. *Multivariate Behav. Res.* 1 245–276. 10.1207/s15327906mbr0102_10 26828106

[B14] Cooper S. E. Martin J. H. Ghez C. (2000). Effects of inactivation of the anterior interpositus nucleus on the kinematic and dynamic control of multijoint movement. *J. Neurophysiol.* 84 1988–2000. 11024092 10.1152/jn.2000.84.4.1988

[B15] Cunningham D. A. Potter-Baker K. A. Knutson J. S. Sankarasubramanian V. Machado A. G. Plow E. B. (2015). Tailoring brain stimulation to the nature of rehabilitative therapies in stroke: a conceptual framework based on their unique mechanisms of recovery. *Phys. Med. Rehabil. Clin. N. Am.* 26 759–774. 10.1016/j.pmr.2015.07.001 26522911 PMC4630680

[B16] Drew T. (1993). Motor cortical activity during voluntary gait modifications in the cat. I. Cells related to the forelimbs. *J. Neurophysiol.* 70 179–199. 8360715 10.1152/jn.1993.70.1.179

[B17] Drew T. Jiang W. Kably B. Lavoie S. (1996). Role of the motor cortex in the control of visually triggered gait modifications. *Can. J. Physiol. Pharmacol.* 74 426–442. 10.1139/cjpp-74-4-426 8828889

[B18] Drew T. Kalaska J. Krouchev N. (2008). Muscle synergies during locomotion in the cat: a model for motor cortex control. *J. Physiol.* 586 1239–1245. 10.1113/jphysiol.2007.146605 18202098 PMC2375657

[B19] Flumerfelt B. A. Otabe S. Courville J. (1973). Distinct projections to the red nucleus from the dentate and interposed nuclei in the monkey. *Brain Res.* 50 408–414. 10.1016/0006-8993(73)90742-7 4196194

[B20] Geed S. Mccurdy M. L. Van Kan P. L. E. (2011). Primate magnocellular red nucleus (RNm) encodes muscle synergies during reaching to grasp. *Soc. Neurosci. Abstr.* 37.

[B21] Geed S. Mccurdy M. L. Van Kan P. L. E. (2013). Contribution of interposito-rubrospinal pathway to muscle synergies underlying reaching to grasp. *Soc. Neurosci. Abstr.* 39.

[B22] Geed S. Van Kan P. L. E. (2016). Grasp-Based Functional Coupling Between Reach- and Grasp-Related Components of Forelimb Muscle Activity. *J. Mot. Behav.* 48 1–17. 10.1080/00222895.2016.1204265 27589010 PMC5733147

[B23] Gentner R. Classen J. (2006). Modular organization of finger movements by the human central nervous system. *Neuron* 52 731–742. 10.1016/j.neuron.2006.09.038 17114055

[B24] Gibson A. R. Horn K. M. Stein J. F. Van Kan P. L. E. (1996). Activity of interpositus neurons during a visually guided reach. *Can. J. Physiol. Pharmacol.* 74 499–512. 10.1139/y96-039 8828895

[B25] Gibson A. R. Horn K. M. Van Kan P. L. E. (1994). “Chapter 5 Grasping Cerebellar Function,” in *Advances in Psychology*, eds Keree M. B. B. Umberto C. (Cambridge, MA: Academic Press), 85–108.

[B26] Holdefer R. N. Miller L. E. (2002). Primary motor cortical neurons encode functional muscle synergies. *Exp. Brain Res.* 146 233–243. 10.1007/s00221-002-1166-x 12195525

[B27] Holstege G. Blok B. F. Ralston D. D. (1988). Anatomical evidence for red nucleus projections to motoneuronal cell groups in the spinal cord of the monkey. *Neurosci. Lett.* 95 97–101. 10.1016/0304-3940(88)90639-8 2465513

[B28] Houk J. C. Gibson A. R. Harvey C. F. Kennedy P. R. Van Kan P. L. E. (1988). Activity of primate magnocellular red nucleus related to hand and finger movements. *Behav. Brain Res.* 28 201–206. 10.1016/0166-4328(88)90097-6 3382512

[B29] Humphrey D. R. Rietz R. R. (1976). Cells of origin of corticorubral projections from the arm area of primate motor cortex and their synaptic actions in the red nucleus. *Brain Res.* 110 162–169. 10.1016/0006-8993(76)90217-1 819107

[B30] Jeannerod M. (1981). “Intersegmental coordination during reaching at natural visual objects,” in *Attention and Performance IX*, eds Long J. Baddeley A. (Hillsdale, NJ: Erlbaum), 153–169.

[B31] Jeannerod M. (1984). The timing of natural prehension movements. *J. Mot. Behav.* 16 235–254. 10.1080/00222895.1984.10735319 15151851

[B32] Johnson M. T. V. Mason C. R. Ebner T. J. (2001). Central processes for the multiparametric control of arm movements in primates. *Curr. Opin. Neurobiol.* 11 684–688. 10.1016/S0959-4388(01)00269-0 11741018

[B33] Jorntell H. Ekerot C. F. (1999). Topographical organization of projections to cat motor cortex from nucleus interpositus anterior and forelimb skin. *J. Physiol.* 514(Pt 2), 551–566. 10.1111/j.1469-7793.1999.551ae.x 9852335 PMC2269074

[B34] Kaiser H. (1974). An index of factorial simplicity. *Psychometrika* 39 31–36. 10.1007/BF02291575

[B35] Kennedy P. R. Gibson A. R. Houk J. C. (1986). Functional and anatomic differentiation between parvicellular and magnocellular regions of red nucleus in the monkey. *Brain Res.* 364 124–136. 10.1016/0006-8993(86)90993-5 3947959

[B36] Krouchev N. Drew T. (2013). Motor cortical regulation of sparse synergies provides a framework for the flexible control of precision walking. *Front. Comput. Neurosci.* 7:83 10.3389/fncom.2013.00083 PMC3708143 23874287

[B37] Kuypers H. G. Fleming W. R. Farinholt J. W. (1962). Subcorticospinal projections in the rhesus monkey. *J. Comp. Neurol.* 118 107–137. 10.1002/cne.901180109 14461005

[B38] Kuypers H. G. J. M. (1982). “A new look at the organization of the motor system,” in *Progress in Brain Research*, eds Kuypers H. G. J. M. Martin G. F. (Amsterdam: Elsevier), 381–403. 10.1016/S0079-6123(08)64138-2 6818612

[B39] Lang C. E. Bastian A. J. (1999). Cerebellar subjects show impaired adaptation of anticipatory EMG during catching. *J. Neurophysiol.* 82 2108–2119. 10561391 10.1152/jn.1999.82.5.2108

[B40] Lawrence D. G. Kuypers H. G. (1968a). The functional organization of the motor system in the monkey. I. The effects of bilateral pyramidal lesions. *Brain* 91 1–14. 4966862 10.1093/brain/91.1.1

[B41] Lawrence D. G. Kuypers H. G. (1968b). The functional organization of the motor system in the monkey. II. The effects of lesions of the descending brain-stem pathways. *Brain* 91 15–36. 4966860 10.1093/brain/91.1.15

[B42] Martin J. H. Cooper S. E. Hacking A. Ghez C. (2000). Differential effects of deep cerebellar nuclei inactivation on reaching and adaptive control. *J. Neurophysiol.* 83 1886–1899. 10758100 10.1152/jn.2000.83.4.1886

[B43] Mason C. R. Miller L. E. Baker J. F. Houk J. C. (1998). Organization of reaching and grasping movements in the primate cerebellar nuclei as revealed by focal muscimol inactivations. *J. Neurophysiol.* 79 k537–554. 10.1152/jn.1998.79.2.537 9463420

[B44] McCurdy M. L. Gibson A. R. Houk J. C. (1992). Spatial overlap of rubrospinal and corticospinal terminals with input to the inferior olive. *Neuroimage* 1 23–41. 10.1016/1053-8119(92)90005-8 9343555

[B45] McCurdy M. L. Hansma D. I. Houk J. C. Gibson A. R. (1987). Selective projections from the cat red nucleus to digit motor neurons. *J. Comp. Neurol.* 265 367–379. 10.1002/cne.902650306 2447133

[B46] Miller L. E. Van Kan P. L. E. Sinkjaer T. Andersen T. Harris G. D. Houk J. C. (1993). Correlation of primate red nucleus discharge with muscle activity during free-form arm movements. *J. Physiol.* 469 213–243. 10.1113/jphysiol.1993.sp019812 8271199 PMC1143869

[B47] Morton S. M. Bastian A. J. (2004). Cerebellar control of balance and locomotion. *Neuroscientist* 10 247–259. 10.1177/1073858404263517 15155063

[B48] Morton S. M. Bastian A. J. (2007). Mechanisms of cerebellar gait ataxia. *Cerebellum* 6 79–86. 10.1080/14734220601187741 17366269

[B49] Overduin S. A. D’avella A. Carmena J. M. Bizzi E. (2012). Microstimulation activates a handful of muscle synergies. *Neuron* 76 1071–1077. 10.1016/j.neuron.2012.10.018 23259944 PMC3547640

[B50] Overduin S. A. D’avella A. Carmena J. M. Bizzi E. (2014). Muscle synergies evoked by microstimulation are preferentially encoded during behavior. *Front. Comput. Neurosci.* 8:20 10.3389/fncom.2014.00020 PMC3942675 24634652

[B51] Overduin S. A. D’avella A. Roh J. Carmena J. M. Bizzi E. (2015). Representation of muscle synergies in the primate brain. *J. Neurosci.* 35 12615–12624. 10.1523/JNEUROSCI.4302-14.2015 26377453 PMC4571600

[B52] Paulignan Y. Frak V. G. Toni I. Jeannerod M. (1997). Influence of object position and size on human prehension movements. *Exp. Brain Res.* 114 226–234. 10.1007/PL00005631 9166912

[B53] Paulignan Y. Jeannerod M. Mackenzie C. Marteniuk R. (1991a). Selective perturbation of visual input during prehension movements. 2. The effects of changing object size. *Exp. Brain Res.* 87 407–420. 1769391 10.1007/BF00231858

[B54] Paulignan Y. Mackenzie C. Marteniuk R. Jeannerod M. (1991b). Selective perturbation of visual input during prehension movements. 1. The effects of changing object position. *Exp. Brain Res.* 83 502–512. 2026193 10.1007/BF00229827

[B55] Pong M. Horn K. M. Gibson A. R. (2002). Spinal projections of the cat parvicellular red nucleus. *J. Neurophysiol.* 87 453–468. 11784762 10.1152/jn.00950.2000

[B56] Ralston D. D. Milroy A. M. Holstege G. (1988). Ultrastructural evidence for direct monosynaptic rubrospinal connections to motoneurons in *Macaca mulatta*. *Neurosci. Lett.* 95 102–106. 10.1016/0304-3940(88)90640-4 2465507

[B57] Rand M. K. Shimansky Y. Stelmach G. E. Bracha V. Bloedel J. R. (2000). Effects of accuracy constraints on reach-to-grasp movements in cerebellar patients. *Exp. Brain Res.* 135 179–188. 10.1007/s002210000528 11131502

[B58] Rispal-Padel L. Latreille J. (1974). The organization of projections from the cerebellar nuclei to the contralateral motor cortex in the cat. *Exp. Brain Res.* 19 36–60. 10.1007/BF00233394 4813399

[B59] Rosner B. (1983). Percentage points for a generalized ESD many-outlier procedure. *Technometrics* 25 165–172. 10.1080/00401706.1983.10487848

[B60] Roy A. C. Paulignan Y. Meunier M. Boussaoud D. (2006). Prehension movements in the macaque monkey: effects of perturbation of object size and location. *Exp. Brain Res.* 169 182–193. 10.1007/s00221-005-0133-8 16328312

[B61] Shinoda Y. Futami T. Mitoma H. Yokota J. (1988). Morphology of single neurones in the cerebello-rubrospinal system. *Behav. Brain Res.* 28 59–64. 10.1016/0166-4328(88)90076-9 3382520

[B62] Shinoda Y. Ghez C. Arnold A. (1977). Spinal branching of rubrospinal axons in the cat. *Exp. Brain Res.* 30 203–218. 202474 10.1007/BF00237251

[B63] Shinoda Y. Yokota J.-I. Futami T. (1982). Morphology of physiologically identified rubrospinal axons in the spinal cord of the cat. *Brain Res.* 242 321–325. 10.1016/0006-8993(82)90316-X 6180798

[B64] Sybirska E. Gorska T. (1980). Effects of red nucleus lesions on forelimb movements in the cat. *Acta Neurobiol. Exp. (Wars)* 40 821–841. 7234514

[B65] Takenobu Y. Hayashi T. Moriwaki H. Nagatsuka K. Naritomi H. Fukuyama H. (2014). Motor recovery and microstructural change in rubro-spinal tract in subcortical stroke. *Neuroimage* 4 201–208. 10.1016/j.nicl.2013.12.003 24432247 PMC3891492

[B66] Van Kan P. L. E. Gibson A. R. Houk J. C. (1993a). Movement-related inputs to intermediate cerebellum of the monkey. *J. Neurophysiol.* 69 74–94. 8433135 10.1152/jn.1993.69.1.74

[B67] Van Kan P. L. E. Houk J. C. Gibson A. R. (1993b). Output organization of intermediate cerebellum of the monkey. *J. Neurophysiol.* 69 57–73. 8433134 10.1152/jn.1993.69.1.57

[B68] Van Kan P. L. E. Horn K. M. Gibson A. R. (1994). The importance of hand use to discharge of interpositus neurones of the monkey. *J. Physiol.* 480(Pt 1), 171–190. 10.1113/jphysiol.1994.sp020351 7853221 PMC1155788

[B69] Van Kan P. L. E. McCurdy M. L. (2001). Role of primate magnocellular red nucleus neurons in controlling hand preshaping during reaching to grasp. *J. Neurophysiol.* 85 1461–1478. 11287470 10.1152/jn.2001.85.4.1461

[B70] Van Kan P. L. E. McCurdy M. L. (2002a). Contribution of primate magnocellular red nucleus to timing of hand preshaping during reaching to grasp. *J. Neurophysiol.* 87 1473–1487. 11877520 10.1152/jn.00038.2001

[B71] Van Kan P. L. E. McCurdy M. L. (2002b). Discharge of primate magnocellular red nucleus neurons during reaching to grasp in different spatial locations. *Exp. Brain Res.* 142 151–157. 10.1007/s00221-001-0924-5 11797092

[B72] Zackowski K. M. Thach W. T. Jr. Bastian A. J. (2002). Cerebellar subjects show impaired coupling of reach and grasp movements. *Exp. Brain Res.* 146 511–522. 10.1007/s00221-002-1191-9 12355280

