# Supplementary material for: Neuronal Correlates of Functional Coupling between Reach- and Grasp-Related Components of Muscle Activity
Source: Front Neural Circuits. 2017 Feb 21;11:7. doi: 10.3389/fncir.2017.00007 (PMC5318413; doi:10.3389/fncir.2017.00007)
Supplement: Supplementary file 1 [file Table_1.DOCX]

Supplementary Material

# Neuronal Correlates of Functional Coupling between Reach- and Grasp-Related Components of Muscle Activity

Shashwati Geed^1, 2, 3*^, Martha L. McCurdy^1^, Peter L. E. van Kan^1^

*** Correspondence:** Shashwati Geed: sg1075@georgetown.edu

# Supplementary Figures and Tables

*Supplementary Table 1. Cumulative variance accounted for by NI, RNm, and muscle components*. Values represent percent variance accounted for (% VAF).

|  | Monkey *W* | | Monkey *B* | |
| --- | --- | --- | --- | --- |
| NI component | Whole-hand | Precision | Whole-hand | Precision |
| 1 | 21.8 | 23.2 | 29.2 | 30.4 |
| 2 | 37.3 | 42.8 | 49.5 | 51.4 |
| 3 | 52.1 | 56.3 | 69.5 | 67.7 |
| 4 | 64.9 | 67.6 | 77.8 | 78.1 |
| 5 | 74.8 | 76.2 | 85.6 | 86.3 |
| 6 | 83.0 | 83.8 | 92.9 | 89.9 |
| 7 | 89.7 | 90.4 |  | 93.4 |
|  | Monkey *W* | | Monkey *B* | |
| RNm component | Whole-hand | Precision | Whole-hand | Precision |
| 1 | 30.5 | 32.8 | 26.8 | 42.8 |
| 2 | 46.7 | 57.0 | 44.2 | 65.4 |
| 3 | 59.1 | 69.4 | 61.5 | 76.7 |
| 4 | 71.1 | 78.1 | 74.3 | 86.7 |
| 5 | 79.7 | 85.0 | 85.4 | 89.9 |
| 6 | 87.0 |  | 91.9 |  |
|  | Monkey *W* | | Monkey *B* | |
| Muscle component | Whole-hand | Precision | Whole-hand | Precision |
| 1 | 29.4 | 35.7 | 27.6 | 38.5 |
| 2 | 54.6 | 56.0 | 54.1 | 65.0 |
| 3 | 73.2 | 75.4 | 74.8 | 76.9 |
| 4 | 90.1 | 86.9 | 84.4 | 84.9 |
| 5 |  |  | 90.2 | 91.8 |
| 6 |  |  | 93.0 |  |
